# Prevalence, Clinico-Laboratory Features and Outcomes of Paediatric Scrub Typhus Cases in a Tertiary Care Centre in Eastern India: A prospective observational study

**DOI:** 10.18295/squmj.6.2024.036

**Published:** 2024-08-29

**Authors:** Raghunath Murmu, Gobinda Mondal, Koushik Biswas, Ashok K. Bala

**Affiliations:** 1 Department of Paediatric Medicine, Midnapore Medical College and Hospital, Midnapore, India; 2 Department of Paediatric Medicine, Dr. B.C. Roy Post Graduate Institute of Paediatric Sciences, Kolkata, India; 3 Department of Biochemistry, All India Institute of Medical Sciences, Raebareli, India

**Keywords:** Scrub Typhus, Rickettsiaceae Infections, Pediatrics, Fever, Enzyme-Linked Immunosorbent Assay, Doxycycline, India

## Abstract

**Objectives:**

Scrub typhus is the most common rickettsial disease in India, caused by *Orientia tsutsugamushi* and transmitted by chigger mites. Previously prevalent in South India, a resurgence of scrub typhus cases has recently affected Eastern India. This study aimed to estimate the prevalence and describe the clinico-laboratory profile of scrub typhus in paediatric patients (1–12 years old) living in Eastern India.

**Methods:**

This prospective observational study was conducted from January to December 2019 at the Dr B C Roy Post Graduate Institute of Paediatric Sciences, Kolkata, India. All acute undifferentiated cases of febrile illness, in patients aged between 1–12 years, were tested using scrub typhus serology by ELISA. Demographic details, clinical features, laboratory findings, complications and treatment outcomes of these scrub typhus patients were extracted and analysed.

**Results:**

Out of 1,473 patients with acute febrile illness, 67 (4.5%) children were diagnosed with scrub typhus. The mean age of the selected patients was 5.22 ± 3.05 years, and the majority (64.2%) had been running a fever since the preceding 7–14 days. Gastrointestinal symptoms such as vomiting (43.3%) and abdominal pain (32.8%) were most frequently observed. Major clinical signs of scrub typhus were hepatomegaly (41.8%) and splenomegaly (31.3%). Complications were observed in 74.6% of patients, with thrombocytopenia (40.3%) and meningoencephalitis (29.9%) occurring more frequently. The case fatality rate of the study sample was 1.5%.

**Conclusion:**

Classical eschar was absent in three-fourth of the studied patients. Hence, this study advocates laboratory scrub typhus tests for all suspected cases in the endemic region (Eastern India). Prompt treatment with doxycycline and/or azithromycin could prevent complications such as thrombocytopenia/meningoencephalitis and reduce mortality.

**Advances in Knowledge**

*- Compared to previous studies, this study noted a higher incidence of scrub typhus-related complications among children, perhaps due to the delay in their arrival at the tertiary healthcare centre*.
*- To the best of the authors’ knowledge, this study presents the first report of scrub typhus prevalence among children presenting with acute undifferentiated febrile illness in West Bengal, India*.

**Applications to Patient Care**

*- Children with acute undifferentiated febrile illness for over the course of 5 days should be tested for scrub typhus even if they do not present with eschar*.
*- Prominent gastrointestinal symptoms in children with acute undifferentiated febrile illness (lasting for more than 5 days) may be a symptom of scrub typhus*.
*- If testing facilities are not available at a health centre (e.g. those in Community Health Centres), a child with acute undifferentiated febrile illness should be urgently referred to a higher healthcare centre without delay*.

Scrub typhus is caused by the infection of the rickettsial bacteria *Orientia tsutsugamushi*, a small Gram-negative, obligate intracellular organism. *O. tsutsugamushi* is transmitted to humans by the bite of the larvae of the Trombiculite mite (chigger).^1^ Infected chiggers are usually found in areas replete with heavy scrub vegetation during wet seasons.^2^ Scrub typhus is endemic to the ‘tsutsugamushi triangle’, a geographic area confined between South and Southeast Asia, Northern Australia and the islands of the Indian and Pacific Oceans.^3^ Globally, one billion people are at risk of being infected and over one million scrub typhus infections occur each year across the world.^4^ The risk factors related to scrub typhus infection include agricultural work, location of residence (in riverbanks, forest clearings, grassy regions), poor sanitation around the house (which favours rodent infestation), vegetation in the house yard, close contact with domestic animals and poor occupational safety practices.^5^

The World Health Organization (WHO) identifies scrub typhus as an emerging disease in Southeast Asia, with a case fatality rate reaching up to 30% if this disease is left untreated.^6^ In fact, India has witnessed a resurgence of scrub typhus in recent times. This disease leads to acute febrile illness, with symptoms that overlap with other viral and bacterial illnesses; it presents a high level of morbidity and mortality.^7^^,^^8^ Devasagayam *et al*. conducted a systematic review to estimate the burden of scrub typhus across India, reporting that the resurgence of the disease is prominent in the nation’s South Indian, sub-Himalayan and North Indian states.^9^ Unfortunately, there is a lack of adequate data on the prevalence and trends of scrub typhus in paediatric populations in India. The majority of the studies on scrub typhus among India’s paediatric population are retrospective or isolated case reports.^10^ Most parts of India witness a surge in scrub typhus cases from July to November, which corresponds to the nation’s monsoon and post-monsoon seasons.^11^^,^^12^ In South India, some outbreaks of this disease also have occurred across the cooler months (from September to January).^13^

Scrub typhus diagnostic methods are broadly classified into direct and indirect methods. Direct methods include isolation and culture of the bacteria, as well as the diagnosis of the genetic material by polymerase chain reaction (PCR). Although a cell culture facility can be used for the *in vitro* cultivation of the bacteria, this is a time-consuming process that requires a specialised laboratory possessing trained personnel and also has limited clinical utility. On the other hand, molecular methods such as PCR have been developed to detect various genes (56kDa, 47kDa and *groEL* genes) of rickettsia. However, conducting such a test is an expensive process and requires significant personnel training.^14^^,^^15^

Instead, indirect diagnostic methods aim to detect the *O. tsutsugamushi*-specific antibodies which reportedly appear in an affected individual due to humoral immunity. These methods include immunofluorescence assay (IFA), immunochromatographic test (ICT), enzyme-linked immunosorbent assay (ELISA), Weil–Felix test and immunoperoxidase assays.^14^ In fact, IFA is considered the gold standard regarding the diagnosis rickettsial infections. In this test, a mixture of antigens (Kato, Karp, Gilliam and any local serotypes) from the common strains of *O. tsutsugamushi* are usually used to detect antibodies in the patient’s serum. This antigen-antibody complex is subsequently detected using a fluorescently labelled anti-human antibody.^16^^,^^17^ In the USA, indirect immunofluorescence antibody test for scrub typhus diagnosis is available in most public laboratories.^18^ Although this test is expensive and requires considerable human resource training, the fluorescence microscope required to carry out this test is available at limited centres in developing countries such as India. On its part, the Weil-Felix test, the most widely used serological test for rickettsial screening, demonstrably has low levels of sensitivity and specificity.^19^ On the other hand, ELISA kits can rapidly detect scrub typhus antigen-specific IgM or IgG antibodies; these kits use the *O. tsutsugamushi* recombinant p56kD type-specific antigen of Karp, Kato, Gilliam and TA716 strains, showing over 90% sensitivity and specificity with regard to detecting scrub typhus-specific antibodies in blood.^20^ Furthermore, point of care testing has also been recently developed for the detection of scrub typhus.^15^

Acute febrile illness is a very common presentation in children living in tropical countries. Diagnosis is often challenging as different paediatric infectious diseases tend to have common symptoms.^21^ Acute febrile illness clinically manifests as a non-specific febrile illness, accompanied by myalgia, headache and occasional rashes, being often associated with gastrointestinal, respiratory or central nervous system symptoms. In these cases, lack of treatment may lead to severe multi-organ dysfunction. However, identifying a case of paediatric scrub typhus is challenging due to the presentation of various confusing symptoms, scarce knowledge about the disease and the low index of suspicion among paediatricians.^10^ Fortunately, the availability of scrub typhus-specific ELISA kits at the government-run medical colleges and district hospitals in India are now enabling paediatricians to rapidly detect scrub typhus in children with acute febrile illness. Early identification and treatment of this disease with doxycycline and/or azithromycin reportedly prevents further complications and improves patient outcomes.^22^ In this context, the present study aimed to estimate the prevalence and describe the clinico-laboratory profile of scrub typhus in paediatric patients (1–12-year-old) in Eastern India.

## Methods

This prospective observational study was conducted from January to December 2019 at the Dr B C Roy Post Graduate Institute of Paediatric Sciences, Kolkata, India, a tertiary care centre in Eastern India. This study included children between 1–12 years of age with acute undifferentiated febrile illness exceeding 5 days in terms of duration, children who were admitted in the paediatric ward, whose scrub typhus IgM ELISA test results was positive and those who ordinarily resided in the Indian state of West Bengal. Children showing acute undifferentiated febrile illness clinically suggestive of scrub typhus but were seronegative, those testing positive for blood and/or urine culture, those who were seropositive for dengue and those with congenital heart disease, nephrotic syndrome, chronic liver disease and severe acute malnutrition were excluded. The febrile children presenting incomplete or missing data were also excluded.

Cases of acute undifferentiated febrile illness lasting for or exceeding 5 days (with or without eschar) were suspected as cases of rickettsial infection (if eschar was present, a fever of less than 5 days’ duration was considered indicative of scrub typhus). Moreover, a suspected clinical case with an optical density above 0.5 for scrub typhus IgM by ELISA was also considered a probable case of scrub typhus.

This study used a convenient sampling method.^23^ It calculated the sample size (n) based on the following formula:


$$\text{n}={\left(\text{z-score}\right)}^{2}\times \text{p}\times \text{q}/{\text{e}}^{2}$$

Taking a z-score of 1.645 at a 90% confidence interval, the prevalence (p) of scrub typhus in febrile children (reported by a previous study) as 3.15% and a 1% margin of error (e), a sample size of 826 was calculated.^16^ After considering a non-response rate of 10%, the sample size increased to 909.

Scrub typhus IgM antibodies in patient serum were detected by indirect ELISA using Microlisa^TM^ kits (J. Mitra & Co. Pvt. Ltd., New Delhi, India). As per the literature on these kits, this study’s in-house kit evaluation demonstrated a kit sensitivity of 100% and specificity of 98.58%, while the external evaluation depicted a kit sensitivity of 100% and specificity of 100%. If the scrub typhus IgM units exceeded 11, the given sample was interpreted as positive for scrub typhus IgM antibodies. Those who tested positive for the scrub typhus serology (ELISA test) were included for further analysis.

Based on the objectives of the study, a proforma was pre-designed to record the history, examination findings and investigation reports of the included patients. This pre-designed proforma was used to collect and record (in detail) the participants’ history, including name, age, sex, date of admission, brief history, clinical findings, investigation reports and outcomes. The subsequent investigations included complete blood count, liver function test, renal function test, prothrombin time, activated partial thromboplastin time, urine routine and microscopic examination, chest x-ray, electrocardiogram, echocardiography, ultrasonography of the whole abdomen, cerebrospinal fluid examination (if required) and computed tomography/ultrasound brain (if required). An Excel spreadsheet (Microsoft Inc., Redmond,. Washington, USA) was used to record the main findings of each patient’s data from the pre-designed proforma.

Standard criteria were used to define the various complications of scrub typhus. Anaemia was considered when a patient’s haemoglobin level was found to be less than 11 g/dL, less than 11.5 g/dL and less than 12 g/dL in the 13–59 months, 5–11 years and 12 years of age groups, respectively. A white blood cell count of 4,000–11,000/μL, a platelet count of 150,000–450,000/μL, an erythrocyte sedimentation rate of 0–10 mm/hour (in the 1–12-year-old age group) and a C-reactive protein count of less than 3 mg/L was considered normal. When the rise in a patient’s serum transaminases was found to be more than twice the upper normal limit, liver enzymes were deemed elevated. A urine output less than 500 mL/1.73 m^2^ per day was considered indicative of oliguria. A serum sodium level less than 135 mEq/L was considered indicative of hyponatremia. A Glasgow Coma Scale of 7/15 to 10/15 was considered indicative of an altered sensorium. In turn, an altered sensorium along with signs of meningeal irritation and/or seizures (associated with elevated protein) and lymphocytic/neutrophilic cytology with normal/low CSF sugar was considered indicative of meningoencephalitis. The dysfunction of more than one organ, requiring intervention to maintain homeostasis, was considered indicative of multiple organ dysfunction syndrome.

Strict confidentiality was maintained throughout the study regarding the patient data that were utilised. Continuous data were checked for normality using the Kolmogorov-Smirnov test. The parametric data were presented as mean ± standard deviations, while the non-parametric data were presented as median and interquartile range. All the categorical data were presented in terms of frequency and percentage. These data were analysed using Statistical Package for Social Sciences (SPSS), Version 25 (IBM Corp., Armonk, New York, USA).

Ethical approval for the study was obtained from the Institutional Review Committee before its commencement (Memo No. BCH/ME/PR/2964). A leaflet containing relevant information about the study was provided to the parents of the patients; thereafter, informed consent (in writing) of the parents of all children participating in this study was obtained.

## Results

A total of 1,473 children were reportedly admitted with acute febrile illness. Among them, 67 were confirmed to be IgM-positive for scrub typhus and included in this study. The prevalence of scrub typhus among children admitted with acute febrile illness was 4.5%. The prevalence of scrub typhus was 4.7% in female patients and 4.4% in male patients. However, upon analysing the seropositive cases, it was observed that the disease was more frequent among males (59.7%) compared to females (40.3%). The mean age of the patients was 5.22 ± 3.05 years, with 38 (56.7%) of the patients belonging to the 1–5-year-old age group [Table 1].

Fever affected all 67 (100%) children included in this study. The fever lasted for 6–7 days in 9 (13.4%) children, 7–14 days in 43 (64.2%) children and over 14 days in 15 (22.4%) children. The mean duration of fever in the scrub typhus-positive patients was 10.67 ± 3.90 days. Other symptoms were vomiting (n = 29, 43.3%), abdominal pain (n = 22, 32.8%), dyspnoea (n = 15, 22.4%), cough (n = 13, 19.4%), diarrhoea (n = 13, 19.4%), convulsion (n = 13, 19.4%), altered sensorium (n = 7, 10.4%), oliguria (n = 5, 7.5%) and headache (n = 5, 7.5%). Upon examination, hepatomegaly was observed in 28 (41.8%) children, followed by splenomegaly in 21 (31.3%), oedema in 16 (24.0%), eschar in 16 (24.0%), maculopapular rash in 14 (20.9%), lymphadenopathy in 11 (16.4%), meningeal signs in 8 (11.9%), hypotension in 8 (11.9%) and icterus in 4 (5.9%) children [Table 2].

Anaemia was observed in 44 (65.7%) children, leukocytosis in 35 (52.2%) children, thrombocytopenia in 27 (40.2%) children, raised erythrocyte sedimentation rate in 47 (69.1%) children, raised C-reactive protein levels in 20 (29.9%) children, elevated liver enzymes in 21 (31.3%) children and hyponatremia in 20 (29.9%) children. Furthermore, 16 (23.9%) children presented with an abnormal chest radiography. Whole abdomen ultrasonography gave the impression of hepatomegaly in 35 (52.5%) children, hepatosplenomegaly in 27 (43.3%) children and ascites in 21 (31.3%) children [Table 3].

Out of 67 paediatric patients, 50 (74.6%) developed complications. The most frequent complication was thrombocytopenia in 27 (40.3%) patients and meningoencephalitis in 20 (29.9%) patients. While undergoing treatment, 1 (1.5%) patient died [Table 4].

## Discussion

This study aimed to estimate the prevalence and describe the clinico-epidemiological profile of scrub typhus-positive patients who were hospitalised due to acute febrile illness. Considering the WHO’s declaration of scrub typhus as a re-emergent infectious disease in Southeast Asia with a case fatality rate of 30%, this study deemed it important to identify and initiate the treatment of scrub typhus infection in its early stages.^6^ The majority of the current study’s participants (56.7%) belonged to the 1–5-year-old age group, while the remaining (43.3%) patients belonged to the 5–12-year-old age group; Gurunathan *et al*. and Ganesh *et al*. reported similar findings.^6^^,^^24^ Children in the former age group tend to play outdoors for prolonged periods and are consequently more likely to get exposed to the bacteria that causes scrub typhus. Moreover, the sex ratio of scrub typhus patients in the current study was 1.48:1. Similarly, Kumar Bhat *et al*. and Basu *et al*. also reported that this disease was more frequent among male children.^25^^,^^26^ By way of explanation, social customs in most parts of India allow boys to play outdoor games while girls stay indoors.^27^ During outdoor play, male children are more likely to be infected by the chiggers. The higher frequency of scrub typhus infection among male children is also reported by studies conducted in Thailand and Taiwan.^28^^,^^29^

The current study observed that the majority (64.2%) of scrub typhus patients reportedly ran a fever for the preceding 7–14 days, with the mean duration of fever amounting to 10.67 ± 3.90 days. Kumar Bhat *et al*., Basu *et al*. and Sah *et al*. reported a similar duration of fever after hospital arrival.^25^^,^^26^^,^^30^ This could be due to patients’ parents suspecting a viral aetiology during the first week of acute febrile illness and therefore not consulted a paediatrician early.

Vomiting (43.3%) and abdominal pain (32.8%) were recorded as the most common presentations associated with scrub typhus-related fever in this study. In this regard, Aung-Thu *et al*. reported that the predominance of gastrointestinal symptoms can enable the differentiation of scrub typhus from other febrile illnesses such as malaria, dengue and leptospirosis.^31^ Moreover, the classical sign of eschar was noted in only 23.9% of scrub typhus patients in the current study. In this vein, Kim *et al*. reported that eschar can be seen in 7–68% of scrub typhus cases.^32^ While the presence of eschar could be a valuable clinical clue, its absence cannot rule out the presence of scrub typhus. The authors of this study suggest that scrub typhus should always be considered a differential diagnosis for patients presenting with acute undifferentiated febrile illness (exceeding 5 days) and gastrointestinal symptoms.

Upon clinical examination, the current study most commonly found the following ailments in its scrub typhus patients: hepatomegaly (41.8%) and splenomegaly (31.3%). Hepatomegaly was reported in 94.7% of patients by Ganesh *et al*., in 82% by Kumar Bhat *et al*. and in 33.3% by Dass *et al*.^24^^,^^25^^,^^33^ Moreover, splenomegaly was reported in 89.9% of patients by Ganesh *et al*., in 39% by Kumar Bhat *et al*. and in 45.8% by Dass *et al*.^24^^,^^25^^,^^33^ In the current study, lymphadenopathy was observed in 16.4% of patients, while it was found in 17.7% of patients in the study by Sah *et al*., in 38% by Kumar Bhat *et al*. and in 59% by Basu *et al*.^25^^,^^26^^,^^30^ These findings suggest that paediatric patients in endemic areas with acute febrile illness exceeding 5 days should be thoroughly screened for hepatomegaly, splenomegaly and lymphadenopathy; such screenings can facilitate the commencement of treatment prior to the arrival of serological reports.

Most patients (74.6%) in the current study developed complications induced by scrub typhus infection. Thrombocytopenia (40.3%) and meningoencephalitis (29.9%) were the most frequent complications among the current study’s participants. In this regard, meningoencephalitis was reported in 58.6% of patients in the study by Lurshay *et al*., in 34.4% by Basu *et al*., in 30.3% by Kumar Bhat *et al*. and in 6% of patients by Palanivel *et al*.^25^^,^^26^^,^^34^^,^^35^ However, the current study reported a higher number of patients with scrub typhus-related complications. This could be attributed to the fact that the institution in this study is a tertiary centre where referral patients from many district hospitals arrive for admission and further treatment. Moreover, the case fatality rate observed by the current study was 1.5%. The sole patient who died was a 7-year-old male child who had presented with fever over 12 preceding days, along with altered sensorium and generalised oedema. After treatment with doxycycline and/or azithromycin, a complete recovery with no post-meningoencephalitis sequelae at the time of discharge was observed in the other patients. In fact, doxycycline is the drug of choice regarding the treatment of scrub typhus. In children, it may be administered either orally or intravenously. For children weighing less than 40 kg, 2.2 mg/kg of body weight, twice daily should be administered. Those weighing over 40 kg should be given 100 mg of doxycycline twice daily. Moreover, the drug should be administered for 3 days after the fever subsides or for a total of 7 days. Severe or complicated cases of scrub typhus may also need antibiotic therapy until 10 days. If fever persists even after 48 hours of starting doxycycline therapy, alternative antibiotics should be initiated or further investigations should be conducted to rule out the possibly of co-infection. Alternative antibiotics which may be prescribed in scrub typhus cases include azithromycin, clarithromycin, chloramphenicol and rifampicin. In paediatric scrub typhus cases, azithromycin is usually given for 5 days (at the rate 10 mg/kg of body weight per day).^36^^,^^37^

The current study was subject to certain limitations. First, as the study was conducted in a tertiary-level referral hospital, its results might not reflect the actual burden of scrub typhus in the surrounding urban communities. Second, the aforementioned hospital is located in a metropolitan city in Eastern India; as chiggers are found more frequently in shrubs and bushes, this disease is likely to be more prevalent in the rural areas of West Bengal. Finally, owing to the improvements of rural district hospitals in the recent years and the availability of ELISA-based scrub typhus kits in such hospitals, many new scrub typhus cases may now be detected and managed there. Such a shift may lead to an underestimation of scrub typhus’ burden in hospitals located in metropolitan cities. It should be noted that this study used IgM ELISA kits to detect scrub typhus, rather than using indirect immunofluorescence assay, which is considered the gold standard in scrub typhus detection.

## Conclusion

Scrub typhus is considered an emerging cause of febrile illness among children in Eastern India. In this study, the classical eschar indicating the presence of scrub typhus was not present in three-quarters of the studied patients; hence, for all cases of acute febrile illness lasting for over 5 days, this study advocates laboratory tests aimed at detecting scrub typhus. This study also found thrombocytopenia and meningoencephalitis to be prominent scrub typhus-related complications. In such cases, prompt empirical therapy with doxycycline and/or azithromycin should be initiated before serological confirmation; this can prevent life-threatening complications and mortality due to scrub typhus. In cases of this disease’s outbreak, each affected state’s health bodies and local rural/urban bodies should be immediately notified so that they can clean the shrubs and bushes in relevant areas, a step which facilitates the reduction of scrub typhus transmission.

## Figures and Tables

**Table 1:** Age- and gender-wise prevalence of scrub typhus in children with acute febrile illness (N = 67)

Characteristic	Total prevalence, n/subtotal (%)	n (%)
**Age group in years**		
1–5		38 (56.7)
>5–12		29 (43.3)
**Gender**		
Male	40/904 (4.4)	40 (59.7)
Female	27/569 (4.7)	27 (40.3)
Total	67/1,473 (4.5)	

**Table 2:** Clinical characteristics of scrub typhus patients (N = 67)

Symptom	n (%)
**Fever duration in days**	
6–7	9 (13.4)
7–14	43 (64.2)
>14	15 (22.4)
**Clinical characteristic**	
Vomiting	29 (43.3)
Abdominal pain	22 (32.8)
Dyspnoea	15 (22.4)
Cough	13 (19.4)
Diarrhoea	13 (19.4)
Convulsion	13 (19.4)
Altered sensorium	7 (10.4)
Oliguria	5 (7.5)
Headache	5 (7.5)
**Clinical finding**	
Hepatomegaly	28 (41.8)
Splenomegaly	21 (31.3)
Oedema	16 (24.0)
Eschar	16 (24.0)
Maculopapular rash	14 (20.9)
Lymphadenopathy	11 (16.4)
Meningeal signs	8 (11.9)
Hypotension	8 (11.9)
Icterus	4 (5.9)

**Table 3:** Laboratory and radiological abnormalities of scrub typhus patients (N = 67)

Parameter	n (%)
**Anaemia**	44 (65.7)
**Total leukocyte count**	
<4,000	3 (4.5)
4000–11,000	29 (43.3)
>11,000	35 (52.2)
**Platelet count**	
<50,000	3 (4.5)
50,000–100,000	17 (25.4)
100,000–150,000	7 (10.4)
>150,000	40 (59.7)
**Erythrocyte sedimentation rate >10 mm/1st hour**	47 (69.1)
**Raised C-reactive protein**	20 (29.9)
**Elevated liver enzymes**	21 (31.3)
**Hyponatremia**	20 (29.9)
**Abnormal chest x-ray findings**	16 (23.9)
**Ultrasonography whole abdomen impression**	
Hepatomegaly	35 (52.2)
Hepatosplenomegaly	27 (43.3)
Ascites	21 (31.3)

**Table 4:** Complications in scrub typhus patients (N = 67)

Complication	n (%)
Thrombocytopenia	27 (40.3)
Meningoencephalitis	20 (29.9)
Pneumonia	12 (17.9)
Shock	12 (17.9)
Pleural effusion	10 (14.9)
Hepatitis	6 (9.0)
Congestive cardiac failure	6 (9.0)
Acute respiratory distress syndrome	3 (4.5)
Acute kidney injury	2 (3.0)
Multiple organ dysfunction syndrome	2 (3.0)
Pulmonary haemorrhage	1 (1.5)
Disseminated intravascular coagulation	1 (1.5)
Death	1 (1.5)
